# Effect of (−)-Epigallocatechin Gallate to Staphylococcal Enterotoxin A on Toxin Activity

**DOI:** 10.3390/molecules25081867

**Published:** 2020-04-17

**Authors:** Yuko Shimamura, Mio Utsumi, Chikako Hirai, Ami Kurokawa, Toshiyuki Kan, Norio Ohashi, Shuichi Masuda

**Affiliations:** 1 School of Food and Nutritional Sciences, University of Shizuoka, 52-1 Yada, Suruga-ku, Shizuoka 422-8526, Japan; shimamura@u-shizuoka-ken.ac.jp (Y.S.); s16204@u-shizuoka-ken.ac.jp (M.U.); gp1848@u-shizuoka-ken.ac.jp (C.H.); s17205@u-shizuoka-ken.ac.jp (A.K.); ohashi@u-shizuoka-ken.ac.jp (N.O.); 2 Department of Synthetic Organic & Medicinal Chemistry, School of Pharmaceutical Sciences, University of Shizuoka, 52-1 Yada, Suruga-ku, Shizuoka 422-8526, Japan; kant@u-shizuoka-ken.ac.jp

**Keywords:** catechin, (−)-epigallocatechin gallate, staphylococcal enterotoxin A, binding

## Abstract

Staphylococcal enterotoxin A (SEA) functions both as superantigens that stimulate non-specific T cell proliferation as well as potent gastrointestinal toxins. We previously reported that (−)-epigallocatechin gallate (EGCG) binds to SEA. Therefore, the ability of EGCG to inhibit SEA toxin activity was examined. As a result, EGCG significantly decreased SEA-induced expression and production of interferon gamma (IFN-γ). In addition, EGCG inhibited SEA-induced spleen cell proliferation. To investigate the role of the galloyl group in EGCG on SEA cytotoxicity in more detail, the effect of the binding of a hydroxyl group at position 3 of the galloyl group in EGCG to SEA on SEA cytotoxicity was examined using two methylated EGCG. SEA cytotoxicity was significantly controlled in both (−)-3′′-Me-EGCG and (−)-4′′-Me-EGCG. These results suggest that EGCG inhibits toxic activity via direct interaction with SEA or without any interaction with SEA. The binding affinity between SEA and EGCG under in vivo conditions was examined using a model solution. Although after treatment under acidic and alkaline conditions, the presence of protein and the digestive tract model solution, EGCG still interacted with SEA. Our studies are the first to demonstrate the effect of the binding of EGCG to SEA on toxin activity.

## 1. Introduction

*Staphylococcus aureus*, an indigenous bacterium, is sometimes pathogenic to humans and animals [1]. *S. aureus* produces various toxins, among which staphylococcal enterotoxin A (SEA) has superantigenic activity, and is involved in multiple sclerosis, rheumatoid arthritis, psoriasis, atopic dermatitis, and chronic rhinosinusitis [2,3]. Superantigens bind to major histocompatibility complex (MHC) class II molecules on antigen-presenting cells (APCs), activate massive T cells via the T cell receptor (TCR), and consequently produced large amounts of cytokines causing various diseases [2]. SEA crosslinks MHC class II and TCR and shows superantigen activity to induce T cell activity [4,5,6]. Therefore, it is expected that these diseases can be reduced by suppressing the superantigen activity of SEA.

Emetic and superantigen activities [4] have been reported as SEA toxin activities. In vivo testing of SEA emetic activity requires the use of special animals with vomiting centers, such as sunkus and cynomolgus monkeys [7,8]. However, these animals are expensive and very difficult to handle. Therefore, superantigen activity suggested to be associated with emetic activity has been investigated [9]. It has been reported that SEA activates an enormous number of T cells and induces the production of many cytokines, such as interferon gamma (IFN-γ), by its superantigen activity [10,11,12]. 

Catechins are major functional components of green tea (*Camellia sinensis*), and some of these polyphenolic compounds exhibit biological activities, including anticancer, antimicrobial, and antiviral activities [13,14,15]. The four main catechins in green tea are (−)-epicatechin, (−)-epicatechin-gallate, (−)-epigallocatechin, and (−)-epigallocatechin gallate (EGCG). Of these four, EGCG is present in the largest quantity, and thus has been used in much of the research [16,17]. A detailed understanding of the molecular properties of catechins may lead to their application in medicine and food chemistry. We previously reported that EGCG binds to SEA [18], indicating that EGCG may have the ability to inhibit MHC class II binding to SEA. However, changes in SEA toxin activity due to the binding of SEA to EGCG have not been clarified.

In this study, inhibitory effects of EGCG on SEA-induced IFN-γ production and T cell proliferation were examined using mouse spleen cells. Furthermore, considering the possibility that EGCG may inhibit SEA toxin activity by binding to the toxin active site, the binding affinity between SEA and the catechins under in vivo conditions was examined.

## 2. Results

### 2.1. Inhibitory Effects of EGCG on SEA-Induced Cytokine Gene Expression and Production

**Figure 1:**
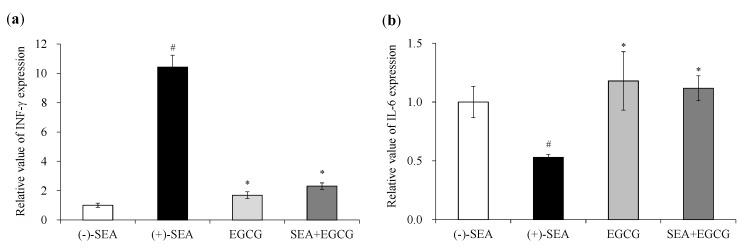
Inhibitory effects of EGCG on staphylococcal enterotoxin A (SEA)-induced cytokine gene expression. (**a**) Gene expression of INF-γ; (**b**) Gene expression of IL-6. ^#^ Represents *p* < 0.05 compared to the (−)-SEA. * Represents *p* < 0.05 compared to the (+)-SEA. Values represent the mean ± SD for three independent experiments.

**Figure 2:**
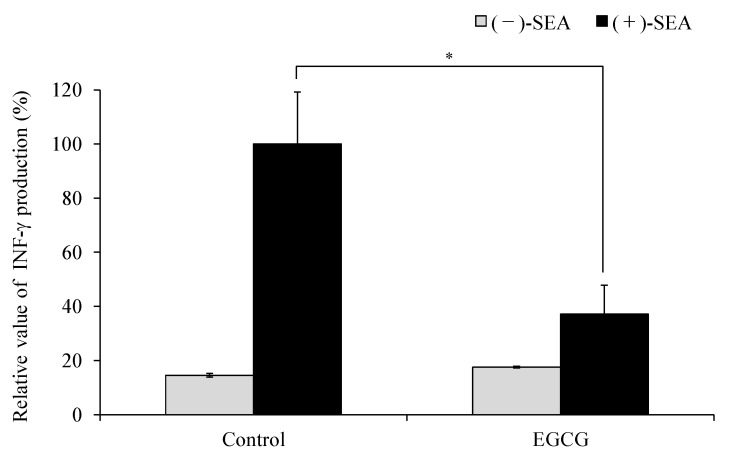
Inhibitory effects of (−)-epigallocatechin gallate (EGCG) on SEA-induced IFN-γ production. IFN-γ production of SEA alone (SEA (+)) was taken as 100%. SEA (100 ng/mL) was used as a positive control. * Represents *p* < 0.05 compared to the SEA (+) control. Values represent the mean ± SD for three independent experiments.

Rasooly et al. (2010) reported that superantigen activity of SEA can be evaluated using mouse spleen cells [18]. The CD4^+^ T cell responses can be generally divided into two distinct types based on cytokine expression, designated Th1 and Th2 [19]. Th1 cells express mainly IFN-γ, whereas Th2 cells express mainly interleukin 6 (IL-6). Therefore, the effects of SEA and SEA + EGCG on the expression of INF and IL-6 gene in mouse spleen cells were examined. SEA and EGCG were added to mouse spleen cells and incubated, and the inhibitory effect on SEA-induced cytokine gene expression and production was measured. As a result, SEA significantly increased the expression of the IFN-γ gene in mouse spleen cells (*p* < 0.05) (Figure 1a). On the other hand, the simultaneous addition of SEA and EGCG to mouse spleen cells significantly inhibited SEA-induced IFN-γ gene expression (*p* < 0.05). Although IL-6 gene expression in mouse spleen cells was decreased by the addition of SEA, the simultaneous addition of SEA and EGCG to mouse spleen cells caused a return to the same level as the control value (Figure 1b). The amount of IFN-γ production was increased by the addition of SEA, and significantly decreased by the addition of EGCG (*p* < 0.05) (Figure 2).

### 2.2. Inhibitory Effects of EGCG on SEA-Induced the Cell Proliferation

**Figure 3:**
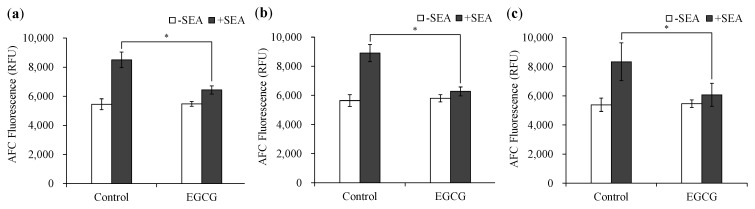
Inhibitory Effects of EGCG on SEA-induced cell proliferation of spleen cells. (**a**) EGCG and SEA were simultaneously added to the spleen cells, (**b**) EGCG after forming a complex with SEA was added to the spleen cells, and (**c**) EGCG was added to the cells and incubated for four hours, and then SEA was added. * Represents *p* < 0.05 compared to the (+)-SEA. Values represent the mean ± SD for the three independent experiments.

The cell proliferation of spleen cells induced by SEA (100 ng/mL) was examined using the glycyl-phenylalanyl-aminofluorocoumarin (GF-AFC) assays. EGCG was added to the spleen cells under three different conditions to examine the effect of EGCG on inhibiting SEA-induced cell proliferation. The three conditions used were as follows: (a) EGCG and SEA were added simultaneously, (b) EGCG after forming a complex with SEA was added to the spleen cells, and (c) EGCG was added to the cells and incubated for four hours, and then SEA was added. As a result, when EGCG and SEA were added simultaneously, and EGCG and SEA were added after binding, EGCG inhibited SEA-induced spleen cell proliferation (Figure 3a,b). Moreover, when SEA was added after the addition of EGCG, EGCG inhibited SEA-induced spleen cell proliferation (Figure 3c).

### 2.3. Effect of Methylated EGCG on SEA Cytotoxicity

**Figure 4:**
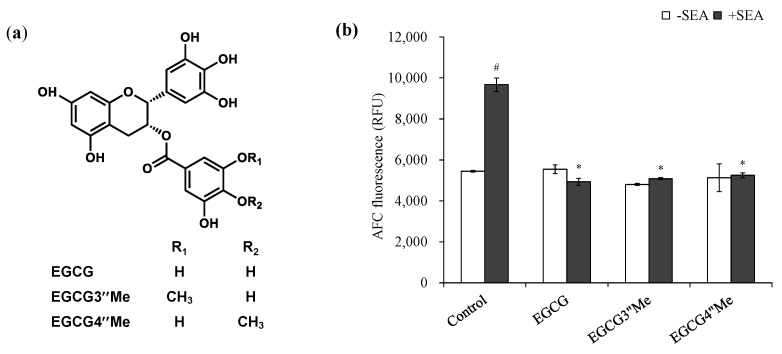
Effect of Methylated EGCG on SEA Cytotoxicity. (**a**) Structure of methylated EGCG, (**b**) Inhibitory effects of methylated EGCG on SEA-induced cell proliferation of spleen cells. ^#^ Represents *p* < 0.05 compared to the (−)-SEA. * Represents *p* < 0.05 compared to the (+)-SEA. Values represent the mean ± SD for the three independent experiments.

We have previously reported the hydroxyl group at position 3 of the galloyl group in the catechin structure was responsible for binding affinity with the Y91 of the A-6 region of SEA active sites. To investigate the role of the galloyl group on SEA cytotoxicity in more detail, the binding effect of hydroxyl group at position 3 of the galloyl group in EGCG to SEA on SEA cytotoxicity was examined using methylated catechins (Figure 4a). In our previous studies, (−)-3′′-Me-EGCG did not bind to SEA. However, SEA cytotoxicity was significantly controlled in both (−)-3′′-Me-EGCG and (−)-4′′-Me-EGCG (Figure 4b).

### 2.4. Effect of pH and Protein Treatment on Binding of EGCG with SEA

**Figure 5:**
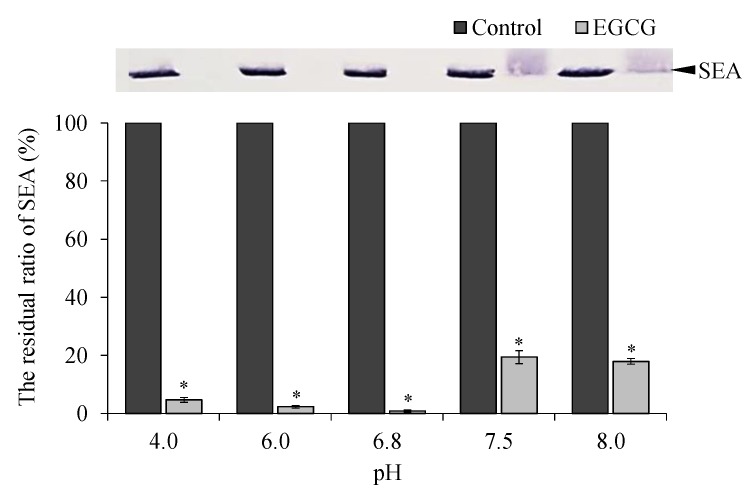
The interaction of EGCG to SEA in various pH conditions. SEA (final concentration 5 μg/mL) and EGCG (final concentration 3.0 mM) in Mcilvaine buffer (pH 4.0, 6.0, 6.8, 7.5, and 8.0) were incubated at 37 °C for 24 h. Following centrifugation, the supernatant was applied to SDS-PAGE and visualized by Western blot analysis. * Represents *p* < 0.05 compared to the (+)-SEA. Values represent the mean ± SD for three independent experiments.

**Figure 6:**
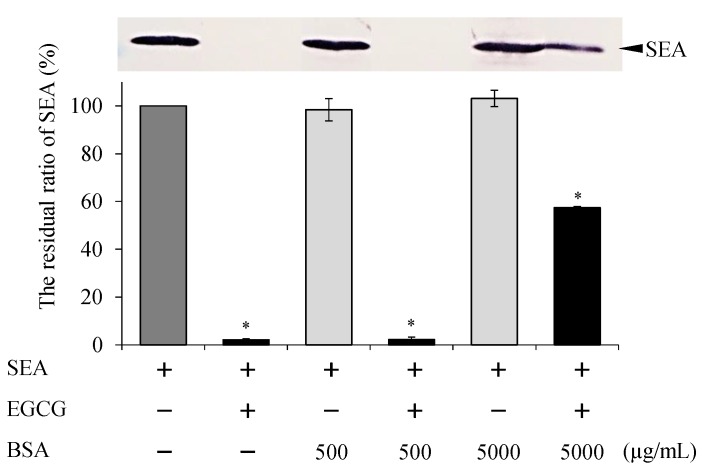
The interaction of EGCG to SEA in the presence of bovine serum albumin (BSA). BSA (final concentration 50, 500, and 5,000 μg/mL) was mixed in a pH 6.8 buffer solution with EGCG (final concentration 3.0 mM), and SEA (final concentration 5 μg/mL) and incubated at 37 °C for 24 h. Following centrifugation, the supernatant was applied to SDS-PAGE and visualized by Western blot analysis. * Represents *p* < 0.05 compared to the (+)-SEA. Values represent the mean ± SD for three independent experiments.

The effect of pH and protein on the binding of EGCG with SEA was examined by Western blot analysis in consideration of the gastrointestinal tract. After treatment under acidic or alkaline conditions (pH 4.0, 6.0, 6.8, and 8.0), EGCG still maintained the binding with SEA (Figure 5). Although the binding of EGCG with SEA was maintained at all pH levels, its strong interaction appeared in the range of pH 6.8 and below. In addition, the interaction between EGCG and SEA was examined. In the presence of BSA protein, no SEA band was detected until the mass concentration ratio of SEA to BSA was 1:100, and the interaction between EGCG and SEA was maintained (Figure 6).

### 2.5. Effect of Pepsin and Pancreatin Treatment on Binding of EGCG with SEA

**Figure 7:**
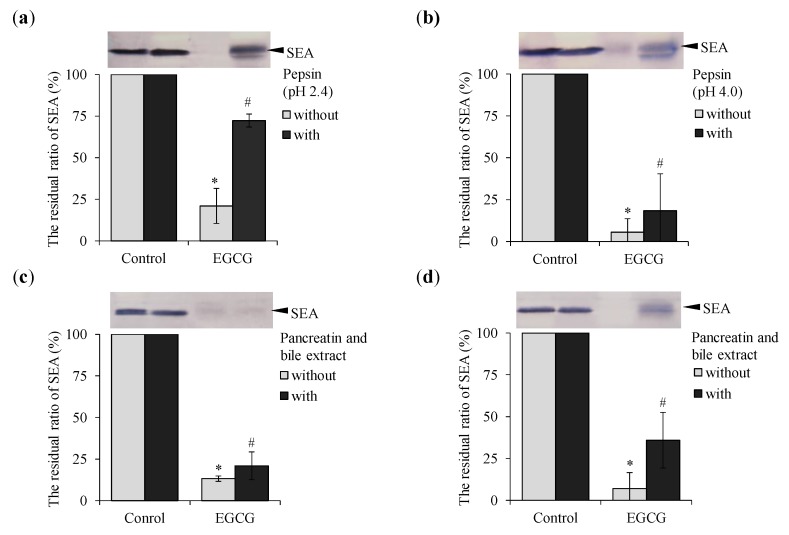
The interaction of EGCG to SEA in the gastrointestinal model solution. (**a**) Pepsin treatment (pH 2.4), (**b**) pepsin treatment (pH 4.0), (**c**) pancreatin and bile extract after the treatment with the pH 2.4 gastric model solution, and (**d**) pancreatin and bile extract after the treatment with the pH 4.0 gastric model solution ^#^ represents *p* < 0.05 compared to the (−)-SEA. * Represents *p* < 0.05 compared to the (+)-SEA. Values represent the mean ± SD for three independent experiments.

It was confirmed whether EGCG interacted even in the gastrointestinal model solutions. As a result, in the intragastric model solution of pepsin treatment at pH 2.4 and 4.0, the binding between EGCG and SEA was slightly inhibited (Figure 7a,b). However, when treated with the intestinal model solution of pancreatin and bile extract after treatment with a gastric model solution at pH 2.4, rebinding of EGCG with SEA was confirmed (Figure 7c). After in vitro treatment with pepsin, followed by pancreatin and bile extract, the binding potency of EGCG with SEA was slightly inhibited (Figure 7d).

## 3. Discussion

SEA activates large numbers of T cells by cross-linking MHC class II and TCR, resulting in excessive cytokine production [10,11,12]. We previously reported that EGCG binds to SEA [18], therefore, it was thought that EGCG may have the ability to inhibit MHC class II binding to SEA. However, changes in SEA toxin activity due to the binding of SEA to EGCG have not been clarified. Rasooly et al. (2010) reported that superantigen activity of SEA can be evaluated using mouse spleen cells [19]. Therefore, the effects of EGCG on the expression of SEA-induced cytokine gene expression in mouse spleen cells were examined. SEA induced IFN-γ (Figure 1a) and suppressed the expression of IL-6 (Figure 1b). These results suggest that SEA induces Th1 cytokines in mouse spleen cells. On the other hand, EGCG significantly inhibited SEA-induced IFN-γ expression and production (Figure 1a and Figure 2). Thus, the inhibitory effect of EGCG on SEA-induced cell proliferation of spleen cells under three different conditions was examined. SEA cytotoxicity activity was evaluated under three conditions by changing the timing of adding EGCG to mouse spleen cells with SEA. As a result, when EGCG and SEA were added simultaneously, EGCG inhibited SEA-induced spleen cell proliferation (Figure 3a). In addition, EGCG and SEA complex inhibited SEA-induced spleen cell proliferation (Figure 3b). The addition of SEA after the addition of EGCG also significantly inhibited mouse spleen cell proliferation (Figure 3c). The point is that SEA activity inhibited under all of the conditions (before, simultaneously, and after) when adding EGCG to SEA. It was reported that apple polyphenols inhibit the binding of SEA between APCs and TCR, and inhibited SEA-induced T cell proliferation in mouse spleen cells [19]. Previous studies have shown that the low polymerized apple procyanidins may inhibit the toxic activity without interaction with SEA [20]. These results suggest that EGCG inhibits SEA toxic activity with or without direct interaction with SEA. EGCG may also inhibit SEA binding between APCs and TCR. 

However, it has not been confirmed that EGCG and SEA bound under the used conditions in this study. Therefore, whether the binding between EGCG and SEA was the essential factor for inhibiting the cytotoxicity of SEA was examined. Previous studies have shown that EGCG and (−)-4-Me-EGCG interact with SEA, and (−)-3′′-Me-EGCG does not interact with SEA [18]. The only difference between EGCG and (−)-3′′-Me-EGCG is the presence of a methyl group at the 3′′-position of the galloyl group (Figure 4a). This information may mean that a hydroxyl group at the position 3 of the galloyl group in the catechin structure was responsible for the binding affinity with the SEA. To confirm that the effect of EGCG binding to SEA on the inhibition of SEA activity, the effect of methylated catechins ((−)-3′′-Me-EGCG and (−)-4′′-Me-EGCG (Figure 4a) on SEA-induced T cell proliferation in mouse spleen cells was examined. As a result, SEA cytotoxicity was significantly inhibited in both (−)-3′′-Me-EGCG and (−)-4′′-Me-EGCG (Figure 4b). These results suggest that binding to SEA is not essential for inhibition of toxin activity. The putative mechanisms of inhibition of SEA toxin activity may be that EGCG and SEA form a complex, or that EGCG inhibits the binding between SEA and MHC class II.

Since SEA toxin activity may be inhibited by the binding of SEA and EGCG, it is desirable that the SEA and EGCG maintain the binding in vivo. Therefore, the binding affinity between SEA and EGCG under in vivo conditions was examined using the model solutions. After treatment under acidic and alkaline conditions (pH 4.0, 6.0, 6.8, 7.5, and 8.0), EGCG still interacted with SEA. The optimal interaction strength appeared in the range of pH 6.8 and below (Figure 5). EGCG is accompanied by spontaneous ring opening under physiological conditions, with a chemical half-life of around 45 min at pH 7.4 and 37 °C [21].

EGCG has been shown to bind to salivary proline-rich proteins [14,22], various proteins in human cells such as serum albumin [23,24]. In addition, EGCG has been reported to bind to and aggregate with casein, which is a protein in foods [25]. When the interaction between EGCG and SEA in the presence of BSA was examined using Western blot analysis, SEA band was not detected until the concentration ratio of SEA to BSA was 1:100. Therefore, the interaction between EGCG and SEA was maintained even in the presence of BSA (Figure 6). These results suggest that SEA and EGCG can maintain their interaction after absorption from the intestinal tract, even in the presence of serum albumin. The strength of interaction between human serum albumin (HSA) having high homology to BSA and EGCG or a methylated EGCG was in the order of (−)-3′′-Me-EGCG > (−)-4′′-Me-EGCG > EGCG [26]. In previous studies, the strength of interaction between SEA and EGCG or a methylated EGCG was in the order of EGCG ≥ (−)-4′′-Me-EGCG > (−)-3′′-Me-EGCG [18]. The flavan-3-ol and the galloyl group of EGCG are essential for site-specific, high-affinity binding for serum albumin [27]. These results suggest that EGCG and BSA may interact with the hydroxyl group at position 4 of the galloyl group, and EGCG and SEA may interact with the hydroxyl group at position 3 of the galloyl group.

Ono et al., reported that SEA bound with submucosal mast cells and induced mast cell degranulation and SEA-induced histamine release plays a critical role in the vomiting response [28]. These findings indicate that the dynamics and interactions of EGCG with SEA in the body are important. In order to examine whether EGCG maintained SEA binding even in the gastrointestinal tract, the binding affinity of EGCG and SEA in an artificial gastrointestinal digestive fluid model was examined. When treated with the intestinal model solution of pancreatin and bile extract after the treatment with the pH 2.4 gastric model solution, rebinding of EGCG with SEA was confirmed (Figure 7a,b). However, after in vitro treatment with pepsin (pH 4.0), followed by pancreatin and bile extract, the binding activity of EGCG with SEA was a little inhibited (Figure 7c,d). It was reported that the stability of EGCG decreased with increasing pH [29]. EGCG derivatives such as its epimer GCG or its digallate dimers theasinensins A/D are generated with increasing pH in aqueous solution [30]. Further studies with the individual EGCG derivatives should be done to ascertain which molecules are the main responsible for the observed attenuation SEA protein in Western blot analysis. Since EGCG may undergo structural changes in vivo, further studies are needed on how the EGCG derivative causes SEA protein reduction. In addition, it will be necessary to examine whether the interactions are maintained in vivo using experimental animals.

## 4. Materials and Methods 

### 4.1. Materials

Staphylococcal enterotoxin A (SEA) with a purity above 95% was bought from Toxin Technology (Sarasota, FL, USA). The compound (−)-epigallocatechin-3-gallate (EGCG) with a purity of 90% was purchased from Wako Pure Chemical (Osaka, Japan). Synthesis of methylated EGCG ((−)-3”-Me-EGCG and (−)-4”-Me-EGCG) were performed according to our reported method [31].

### 4.2. Cytokine Gene mRNA Expression 

The isolation of mouse spleen cells from C57BL/6J female mice (nine weeks) was performed as described above [20]. Animal experimental procedures were performed with the approval of the Institutional Animal Care and Use Committees of the University (Permit Number: 175160 and 185185). EGCG stock solutions (300 mM) was diluted in 10% dimethyl sulfoxide and dimethyl sulfoxide. The spleen cells were placed in 96-well plates (1 × 10^6^ cell/mL) in 9.8 mL of Russ-10 medium and 100 µL of 5.0 mM EGCG (final concentration 0.05 mM) was added with or without 100 µL of 5 μg/mL SEA (50 ng/mL), followed by incubation at 37 °C in a 5% CO_2_ incubator for 16 h. RNA extraction was performed using a RNeasy mini Kit (QIAGEN, Tokyo, Japan), according to the manufacturer’s instructions. The real-time PCR was performed using a SYBR Premix Ex Taq (Takara, Shiga, Japan) and a real time PCR system (Thermal Cycler Dice^®^ Real Time System Single; Takara). Each sample was normalized to hypoxanthine-guanine phosphoribosyl transferase (Hprt), triplicates were averaged, and relative mRNA levels were determined. The primer pairs of IFN-γ were 5′-CATTGAAAGCCTAGAAAGTCTG-3′ and 5′-CTCATGAATGCATCCTTTTTCG-3′, IL-6 were 5′-GTGGCTAAGGACCAAGACCA-3′ and 5′-ACCACAGTGAGGAATGTCCA-3′, and Hprt were 5′-GTTGGATACAGGCCAGACTTTGTTG-3′ and 5′-GAGGGTAGGCTGGCCTATAGGCT-3′.

### 4.3. IFN-γ Detection

Ninety µL of spleen cells were placed in 96-well plates (1 × 10^6^ cell/mL) in Russ-10 medium and 5 µL of 1.0 mM EGCG (final concentration 0.05 mM) was added with or without 5 µL of 1 μg/mL SEA (50 ng/mL), followed by incubation at 37 °C in a 5% CO_2_ incubator for 72 h. After the incubation, the reaction mixture was centrifuged at 2400× *g* for five minutes at 4 °C, and the supernatants were used as samples. The inhibitory effects of samples on IFN-γ production in spleen cell culture supernatants was estimated with the Mouse IFN-γ Quantikine ELISA (R&D Systems, Inc., Minneapolis, MN, USA) according to the manufacturer’s manuals.

### 4.4. Isolation of Mouse Spleen Cells and SEA Cytotoxicity Assay

Inhibitory effect of EGCG on SEA cytotoxicity activity using the GF-AFC assays was reported by Rasooly et al. (2010), with some modifications [19]. In order to examine the influence of the method of adding the EGCG to the cells, the three conditions were used as follows: (1) EGCG and SEA were simultaneously added to the spleen cells, (2) EGCG after forming a complex with SEA was added to the spleen cells, and (3) EGCG was added to the cells and incubated for four hours, and then SEA was added. Briefly, in the method of (1), 245 µL of spleen cells (1 × 10^5^ cell/mL), 225 µL of Russ-10 medium, 25 µL of 3.0 mM EGCG (final concentration 0.15 mM), and with or without 5 µL SEA (final concentration 100 ng/µL) were mixed in 1.5 mL tubes. After transfer 100 μL of the diluted cell sample to a 96-well plate for fluorescence and followed by incubation at 37 °C in a 5% CO_2_ incubator for 48 h. In the method of (2), 24 μL distilled water, 3.0 μL of 20 μg/mL SEA, and 3.0 μL of 300 mM EGCG were mixed, followed by incubation at 37 °C for four hours (EGCG final concentration 3.0 mM). Fifty µL of this SEA (final concentration 100 ng/µL) and EGCG (final concentration 0.15 mM) reaction mixture, 145 µL of spleen cells (1 × 10^5^ cell/mL), and 140 µL of Russ-10 medium were mixed in 1.5 mL tubes. After transfer 100 μL of the diluted cell sample to a 96-well plate for fluorescence and followed by incubation at 37 °C in a 5% CO_2_ incubator for 48 h. In the method of (3), 17 µL of 3.0 mM EGCG, 145 µL of spleen cells (1 × 10^5^ cell/mL), and 138 µL of Russ-10 medium were mixed in 1.5 mL tubes. After transfer 90 μL of the diluted cell sample to a 96-well plate for fluorescence and followed by incubation at 37 °C in a 5% CO_2_ incubator for four hours. After the incubation, 10 μL of 1.0 μg/mL SEA was added (SEA final concentration 100 ng/mL and EGCG final concentration 0.15 mM), and the mixture was incubated at 37 °C in a 5% CO_2_ incubator for 44 h. Distilled water was used for control instead of the EGCG sample. In each condition, the cytotoxicity induced by SEA with or without EGCG was estimated with the MultiTox-Fluor Multiplex Cytotoxicity Assay (Promega Co., Madison, WI, USA) according to the manufacturer’s manuals. 

### 4.5. Effect of Methylated EGCG on SEA Cytotoxicity

The effect of the binding of the hydroxyl group at position 3 of the galloyl group in the EGCG to SEA on SEA cytotoxicity was examined using methylated EGCG. Then 245 µL of spleen cells (1 × 10^5^ cell/mL), 225 µL of Russ-10 medium, 25 µL of 3.0 mM EGCG or methylated EGCG ((−)-3”-Me-EGCG and (−)-4”-Me-EGCG) (final concentration 150 µM), and with or without 5 µL SEA (100 ng/µL) were mixed in 1.5 mL tubes. After adding 100 μL of the diluted cell sample to a 96-well plate for fluorescence and followed by incubation at 37 °C in a 5% CO_2_ incubator for 48 h. Distilled water was used for control instead of the EGCG or methylated EGCG sample. The cytotoxicity was estimated with the MultiTox-Fluor Multiplex Cytotoxicity Assay (Promega Co.) according to the manufacturer’s manuals. 

### 4.6. Effect of pH and Protein on Binding of SEA and EGCG

In consideration of the intragastric, buffers of pH 4.0, 6.0, 6.8, 7.5, and 8.0 were prepared using Mcilvaine Buffer. To examine the effects of pH on the interaction of EGCG with SEA, EGCG (final concentration 3.0 mM) and SEA (final concentration 5 μg/mL) mixed in each pH buffer solution and incubated at 37 °C for 24 h. To investigate the effect of protein on SEA binding, bovine serum albumin (BSA) (Sigma-Aldrich, St. Louis, MO, USA); final concentration 50, 500, and 5000 μg/mL) was mixed in pH 6.8 buffer solution with EGCG (final concentration 3.0 mM) and SEA (final concentration 5 μg/mL) and incubated at 37 °C for 24 h. After 24 h, the reaction mixture was used for SDS-PAGE and Western blot analysis. The residual ratio of SEA protein was determined by Western blot analysis and quantified using ImageJ software (National Institutes of Health, Bethesda, MD, USA).

### 4.7. Digestive Tract Model 

We examined the effects of hydrolysis by gastrointestinal proteases. EGCG (final concentration 3.0 mM) and SEA mixture (final concentration 5 μg/mL) was adjusted to a pH of 2.4 and 4.0 using Mcilvaine buffer, followed by the addition of pepsin from porcine gastric mucosa (Sigma-Aldrich; final concentration 0.32%), and incubated at 37 °C for three hours. After three hours, the solution was adjusted to a pH of five to six with 10 M NaOH to inactivate pepsin, followed by the addition of pancreatin from porcine pancreas (Sigma-Aldrich; final concentration 0.25%) and bile powder (final concentration 0.4%), and incubated at 37 °C for 24 h. After 24 h, the reaction mixture was used for SDS-PAGE and Western blot analysis. The residual ratio of SEA protein was determined by Western blot analysis and quantified using ImageJ software.

### 4.8. Statistical Analysis 

The results were analyzed using a Student t test or one-way ANOVA, followed by the Dunnett’s test using Microsoft Excel 2016 (Microsoft, Redmond, WA, USA). The significance level was set at *p* < 0.05 and all experiments were replicated at least three times. The Tukey–Kramer test was used to compare differences between groups (*p* < 0.05).
